# A Pedagogy of Digital Materiality: Integrated Design and Robotic Fabrication Projects of the Master of Advanced Studies in Architecture and Digital Fabrication

**DOI:** 10.1007/s44150-022-00040-1

**Published:** 2022-05-13

**Authors:** David Jenny, Hannes Mayer, Petrus Aejmelaeus-Lindström, Fabio Gramazio, Matthias Kohler

**Affiliations:** Chair of Architecture and Digital Fabrication, Department of Architecture, Institute of Technology in Architecture (ITA), ETH Zürich, Zürich, Switzerland

**Keywords:** Computational Design, Robotic Fabrication, Constructive Systems, Sustainable Construction, Complex Material Systems, Design Research and Pedagogy

## Abstract

This paper illustrates the pedagogical approach to teaching computational design and digital fabrication in the Master of Advanced Studies in Architecture and Digital Fabrication. It demonstrates how the introduction of computational design and digital fabrication methods foster a holistic approach to integrate novel material and constructive systems into the design process. Such an integration allows the students to combine digital fabrication techniques with sustainable material processes, taking into account the questions of reversibility, recycling and reuse, and thus designing for a more sustainable construction. In the presented paper, the structure and the curriculum of the MAS programme is introduced and the pedagogical approach of the Integrated Design and Robotic Fabrication Project is demonstrated through four case studies, highlighting their respective teaching strategies in combination with the learning experiences of the students.

## Introduction

Digital technologies in architecture have changed the way we understand and work with materials. Complementing a digital design discussion on forms, a digital materiality evolves through the interplay between digital and material processes in design and construction [16]. At the intersection of this interplay, the industrial robot operates, translating digital data into the real world, as it enacts their materialisation. At the same time, sensors operate in the other direction, capturing real world conditions and translating them into quantitative, digital data. Consequently, material and fabrication systems in general have become a meaningful, if not dominant design driver. The architect, now in his new role as a process designer, departs from working with material only in its industrially pre-processed form as building products and learns to integrate the behaviour of minimally processed, “natural” materials into the design process through code and the adaptability of robotic fabrication methods. While a persistently hierarchical approach has often led to the generation of geometric information being prioritised over its subsequent materialisation, it has been pointed out how employing computation to tap into the intricate and multifaceted design potential latent in the material itself enables the development of material systems that no longer need to be derivatives of long-established and standardised building systems and elements [21]. This approach can also be linked to the idea of soft tolerances where a design does not follow a preconceived logic and is not always predictable but rather emerges from a tension between the strict execution of machine code and the more natural, textured or low-definition features of a material that is shaped [19]. Such an approach allows students to combine digital fabrication with natural material processes, taking into account questions of reversibility, recycling and reuse, and thus designing for more sustainable construction.

What does this new approach enabled by digital technology mean for the skill-sets of architects and for teaching architecture? In the nineteenth century, the emergence of the natural sciences, biology and chemistry, ended the dominance of a purely visual approach to understanding nature. Increasingly intricate theories and models were developed, describing nature as a set of dynamic and complex interrelated processes. Similarly, architecture in the digital age is described through new models of interrelated processes, through algorithms and digital code. The first and most basic skill for the contemporary architect is thus learning how to code. Code controls the robot, which manipulates the material accordingly. At the same time, the dynamic behaviour and characteristics of the material are reflected in the code itself or affect the encoded parameters directly through sensors. The second skill for the architect is thus to learn about material behaviour in relation to a constructive system and its properties. The third skill is then to be able to collaborate within and across disciplines, because material knowledge is far too complex to be entirely understood by a single individual. Finally, the fourth skill is simply to design, meaning the ability to synthesize and merge all the other skills into a productive and coherent process.

Over the past six years, the Master of Advanced Studies in Architecture and Digital Fabrication at ETH Zurich [20] has developed a curriculum addressing these skills. It focuses on 3D printing, robotic fabrication and AR/XR technologies and equips students with the technical, intellectual, and artistic skills to master the digital chain from computational design to the fabrication and assembly of full-scale architectural structures. At the heart of the programme sits the Integrated Design and Robotic Fabrication Project, where students work collaboratively on all aspects of a design, from the initial concept and definition of a material system to the robotic fabrication and assembly of the final structure. This part of the MAS DFAB programme has resulted in a series of built structures which demonstrate how integrating material processes, computational design methods and robotic fabrication can lead to new and highly sustainable constructive systems. In this paper we illustrate our pedagogical approach to teaching computational design and digital fabrication through four projects that were realised within the MAS DFAB programme, the Brick Labyrinth [24], Gradual Assemblies [10], Up-Sticks [11] and Rapid Clay Formations [9, 12–14].

## Methodology

### Structure and curriculum of the master of advanced studies in architecture and digital fabrication

The curriculum of the MAS DFAB is structured in three trimesters that build upon one another. The first trimester Digital Foundations is dedicated to training skills in using digital fabrication tools and methods. As it is a post-graduate programme, courses are designed so that students with different backgrounds and levels of experience can follow and quickly build up knowledge in coding and digital fabrication while developing a conceptual understanding of and architectural sensibility for the technologies at hand.

The second trimester is dedicated to the design and construction of a building scale structure. The Integrated Design and Robotic Fabrication Project enables students to apply the knowledge gained in the first trimester to a large-scale fabrication project, taking architectural, structural and logistic constraints into account. With the whole student group working together, emphasis is placed on collaboration and knowledge-exchange, fostering a culture of teamwork that allows them to move successfully from idea to realisation in just ten weeks.

In the third trimester, students are required to develop an individual master thesis based on and in collaboration with ongoing research projects at the Institute.

Each trimester has its distinct focus, pedagogical challenges, and learning goals, aimed at holistic teaching of advanced methods and technologies at the forefront of digital design and fabrication and their implementation in architecture and construction. While the first trimester builds a common ground of knowledge and skills, the second trimester focuses on real-world problems, bridging between academia and practice. Finally, the third trimester introduces specific research methodologies, allows for individual specialization and prepares students for a career in research. Overall the curriculum prioritises conceptual and technological “fluency” over a “skill based” approach following an understanding that the development of adaptive, foundational skills empowers students to manipulate the medium to their advantage and to handle unintended and unexpected problems when they arise [23]. This paper focuses on the structure and results of the second trimester project.

### Pedagogical approach of the integrated design and robotic fabrication project

The pedagogical approach in the Integrated Design and Robotic Fabrication Project pushes the boundaries of a design research methodology in architecture education on several levels. In the context of this paper the notion of design research builds upon the concept of “learning by doing” originally attributed to John Dewey which describes reflective thinking as an an act of search or investigation directed toward bringing to light further facts which serve to corroborate or to nullify the suggested belief [4]. Here, this idea can be extended to include “learning from materials” as well as “learning by fabrication”, where results of empiric testing and physical prototyping are reflected and integrated into a comprehensive computational design model.

First, all projects start with a completely novel constructive system for which a proof of concept has been previously developed by the tutors. Rather than selecting an established fabrication process and calling for a design proposal, the Integrated Design and Robotic Fabrication Project focuses on the refinement and full-scale implementation of the proposed prototypical material and constructive system in a collaborative effort between students and tutors. This approach interweaves the design and fabrication process development, fostering a culture of creative problem-solving skills.

Second, as students work on the parallel development of a computational design workflow and digital fabrication process, physical prototyping at full-scale, structural testing, and construction planning, they experience first hand the need for an integrative design approach, where interdependencies are reflected in the workflow. The different aspects of the project development inform one another, and certain design intentions can directly influence the development of the material aware fabrication process and its tools and vice versa. Findings from prototyping or material tests inform and define the design and feed directly back into the computational design setup. The role of the designer who exerts his own will is transformed into a role where the designer acts as an interpreter of this performative connection between design and material-technological development.

Third, each project is aimed at a clearly scheduled delivery according to a project brief for a real-world design and building task, including the planning of resources and logistics that come with it. As such, during the design process, the knowledge and time of the collective group need to be effectively allocated and the material and machine availability taken into account. In the Integrated Design and Robotic Fabrication Project, this need is addressed by the development of a custom computational design model integrating the complete project chain, from the conceptual design, structural analysis, and generation of fabrication data to the construction segmentation and sequencing. Each project develops its own algorithmic design model and data that allow students to specify which interfaces are needed to efficiently integrate these different tasks into one parametric workflow.

Lastly, the Integrated Design and Robotic Fabrication Project has a hybrid format, which fundamentally addresses questions regarding constructive design, detailing, and assembling, as well as sustainability and reusability. All in all, students learn to apply their conceptual and technical skills to a concrete building task, learning to deal with reality, resources and with the potentials and challenges of design development as collaborative team work. As such, it facilitates rapid project development and becomes itself a testbed for novel ideas and future research projects. Although each of the case studies presented below investigates a different material and fabrication system, they share a common methodology. By pointing out specific didactic methods and teaching strategies, we illustrate how the pedagogical approach evolves from one project to the next.

## Case studies: material, fabrication, design, and pedagogy

### The Brick Labyrinth

**Fig. 1:**
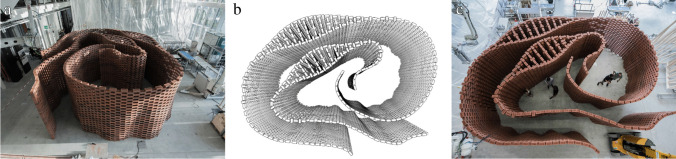
The Bick Labyrinth, a precise definition of a promenade along a sequence of changing spatial and material situations that a visitor can experience by walking through **a**) perspective view, **b**) plan drawing, **c**) top view of the structure

**Fig. 2:**
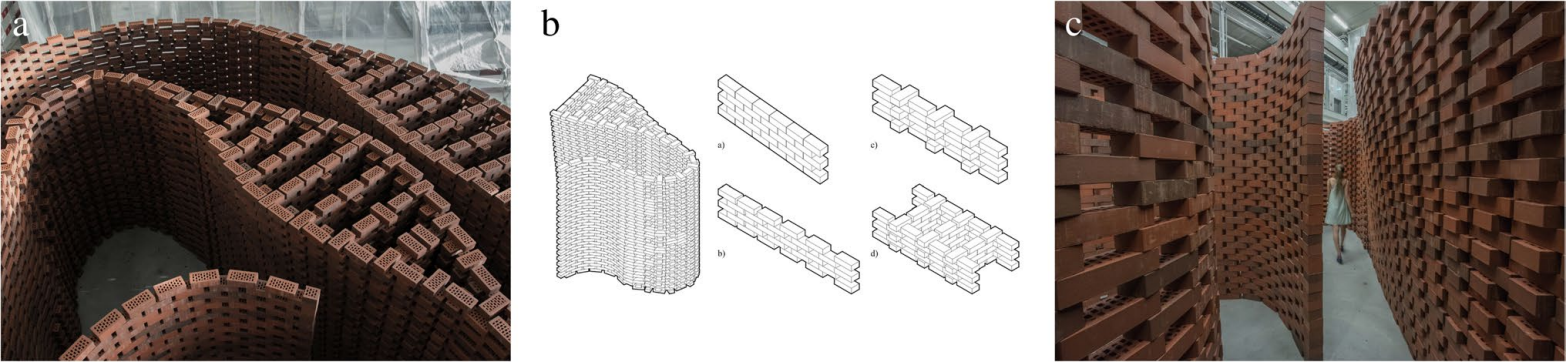
Flexible brick-bond typology, different strategies to increases friction between bricks in a drystacked constructive system allow for smooth transitions between double-curved walls, extreme overhangs, and thin wall sections **a**) close-up view **b**) diagram of the brick bond **c**) impression of the spatial experience

**Fig. 3:**
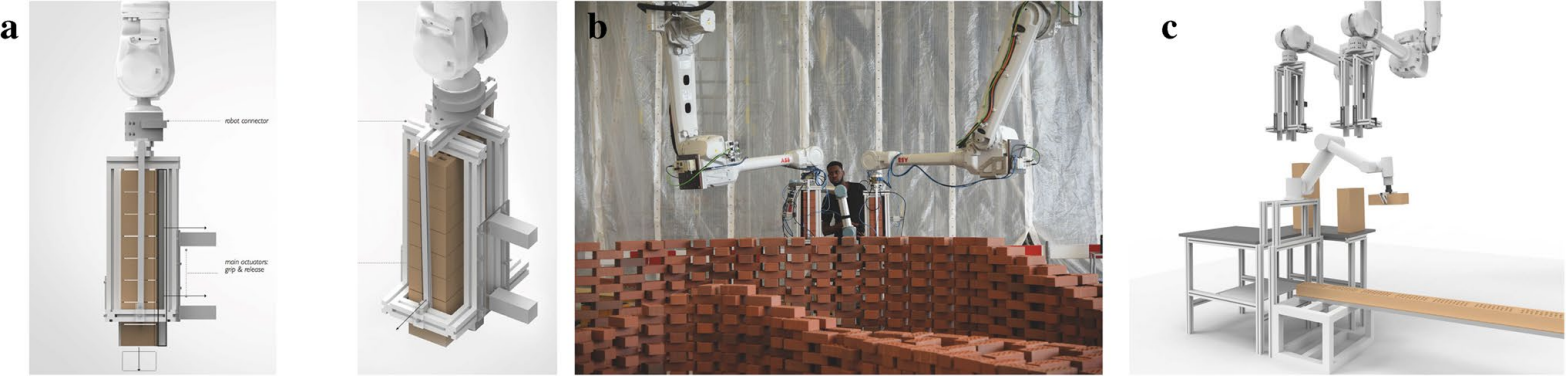
Ecology of robotic systems **a**) diagram of custom end-effector, **b**) demonstration of human–robot collaboration, **c**) and diagram of cooperative robotic process

The Brick Labyrinth is the first iteration of the teaching format of the Integrated Design and Robotic Fabrication Project and as such, a pedagogical experiment. Built as a temporary structure by a multi-robotic process to dry-stack bricks, the Brick Labyrinth is a large-scale, accessible construction providing a unique spatial experience for its visitors (Fig. 1). The design not only proposes an interpretation of a contemporary labyrinth typology, but also develops a specific brick-bond (Fig. 2) and an efficient, fully-reversible digital fabrication process at large-scale (Fig. 3). In parallel to the design development, students simultaneously optimise the robotic setup and mechanical end-effectors, as well as the structural simulation and fabrication procedure, to enable a reliable robotic construction process at high speed [24].

#### Material system, fabrication process, and design

While building on the experience of the previous robotic brick works at Gramazio Kohler Research [2], the Brick Labyrinth increases the significance of computational design and robotic control by working with a dry-stacked construction method (Fig. 2a). The absence of any type of adhesive in the brickwork requires the structure to be in equilibrium at all points in time, and thus the computational design process has to integrate structural simulation and benefits greatly from the potential of the robot to place the bricks with great precision (Fig. 2b). The unique setup of the project and its material system demonstrate a fully reversible construction process at architectural scale, presenting a great potential for teaching because students can physically prototype and spatially experience iterations of their designs at full-scale (Fig. 2c), reusing the same bricks and thus not wasting any material.

The design task is relatively open, as only the maximum amount of bricks and the construction area are given as constraints, but what makes the design process complex is the need to integrate fabrication, structural, and material parameters. This challenge is addressed by a performative collaborative approach, where the division of tasks and definition of interfaces between groups allows students to focus on specific topics. In a first phase, smaller teams are formed to explore different conceptual design approaches to the architectural typology of the labyrinth, which are then collectively evaluated by students and tutors for their spatial potential and feasibility. This process helps to define a common design strategy for the labyrinth: Two differentiated walls spiraling around and leaning towards each other create a series of highly articulated spaces along a path (Fig. 1b, c). Mediated by tutors, students define specific tasks and the team dynamically reorganises into”expert” groups with tasks that include the development of the overall design, the computational design and calculation of structural equilibrium, physical prototyping and testing, as well as fabrication and tooling setup. Although each group works on different topics, the feedback and explorations of the various groups are integrated into one shared computational design model, ensuring informed collaboration and a common learning experience for everyone.

#### Teaching strategy and learning experience: development of an end-effector

The speed of a pick-and-place process decreases as a construction becomes larger, as each brick has to be carried for longer distances. Ultimately, however, the possible size and building time of the Brick Labyrinth directly depend on the speed of the fabrication process. Optimising the process speed thus becomes an important teaching goal that provides the students with the opportunity to develop both the concept and the physical implementation of a robotic process and tooling. Here, the inherently slow robotic pick-and-place procedure was made more efficient by the development of a new robotic end-effector, a brick magazine that can hold a stack of eight bricks at a time and dispense them one after the other (Fig. 3a). While the simultaneous use of two large synchronized robotic arms further increase the speed of the process (Fig. 3b), two small collaborative robotic arms are used to continuously prepare brick stacks to ensure a smooth refill of the end-effectors (Fig. 3c). Students can focus on the material logistics as well as oversee and control the quality of the building process. All in all, this ecology of different robotic systems and components working together with humans and their manual tasks constitutes a holistic approach to developing the design typology, tooling, and fabrication process.

#### Discussion

The Brick Labyrinth demonstrates how a fully reversible digital fabrication process could be used for architectural design explorations in a prototypical setting at full-scale. Conceptually, the reuse of the bricks stands for a completely circular constructive system, where the sustainable use of materials is supported by the efficient and precise execution of the construction and deconstruction, brick by brick from “pallet-to-pallet”. On a didactic level, the design development is closely linked to the further refinement of the tooling, the fabrication process, and the structural performance. The results are evaluated through physical prototypes, and in this way the digital fabrication process itself informs the design idea. Finally, the fabrication process introduces the idea of different robotic systems collaborating with each other and with humans as a powerful alternative to the idea of pure industrial automation.

### Timber Dowel Assemblies: Gradual Assemblies and Up Sticks

**Fig. 4:**
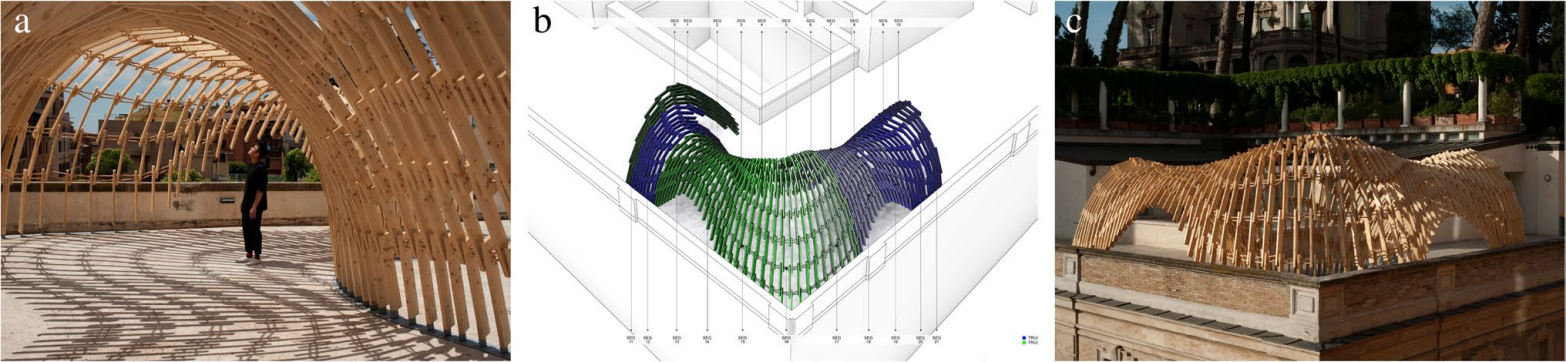
Timber Dowel Assemblies: Gradual Assemblies **a**) spatial experience, **b**) design diagram, **c**) perspective view

**Fig. 5:**
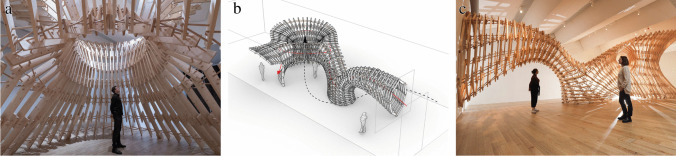
Timber Dowel Assemblies: Up Sticks **a**) spatial experience, **b**) design diagram, **c**) perspective view

**Fig. 6:**
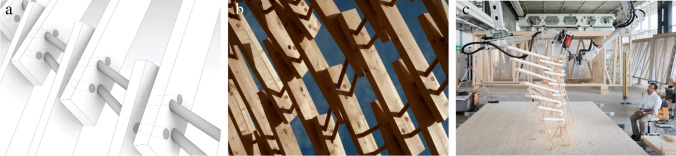
Timber Dowel Assemblies: Development of the joint typology **a**) diagram, **b**) close-up **c**) cooperative robotic fabrication

This section discusses Gradual Assemblies (Fig. 4) and Up Sticks (Fig. 5), the second and third iterations of the Integrated Design and Robotic Fabrication Project, together as they share the development of a common constructive system: Timber Dowel Assemblies, a sustainable timber construction method that utilizes the precision of computational design and robotic fabrication together with the behaviour of a natural material. At the core of the constructive system stands the development of the joint (Fig. 6): Short slats are robotically positioned in space and locked using spatially oriented wood dowels to form a novel timber-only connection, taking advantage of wood’s hygroscopic behaviour.^1^

Prefabricated in segments and assembled as a permanent large-scale structure, Gradual Assemblies applies the constructive system in the design of a sun-shading pergola on the terrace of the Istituto Svizzero in Rome (Fig. 4). The design proposes a single differentiated shell structure that describes a curve along the site, and develops a first simple joint type [10]. Building upon this first implementation, the constructive system is further developed through a MAS Master Thesis exploring its structural behaviour and resulting in the proposal for a second promising joint type. These explorations benefited the second large-scale project, Up Sticks, which was commissioned by the VA Dundee as a temporary exhibition installation to be later re-assembled somewhere else (Fig. 5). The design of Up Sticks pushes the boundaries of the constructive system and refines the connection logic to create an expressive timber structure that twists and curves, providing a stunning spatial experience for the visitors [11]. For each of the projects, students develop a comprehensive computational model based on a network data-structure that integrates the iterative design development and geometry generation as well as a stability check and the segmentation, sequencing, and control of the robotic fabrication as well as on-site assembly process.

#### Material system, fabrication process, and design

Building on the experience of previous robotic timber projects at Gramazio Kohler Research, the material and fabrication system of Timber Dowel Assemblies (TDA) is mainly inspired by two predecessors: The Sequential Roof [1] introduces the idea to use simple and small-profile timber slats that are individually cut to size and assembled through a digitally planned and executed fabrication process to form complex and differentiated structures, in which short timber slats are assembled in layers and connected by simple nail connections. Timber Dowel Assemblies extends this concept to include the potential to position and spatially orient each individual slat, building upon explorations of Complex Timber Structures [17, 18]. Finally, it develops the constructive logic of a new spatial timber-only dowel connection.

Timber Dowel Assemblies as a constructive system introduces novelties that not only expand the design space and geometric freedom but also relate on a conceptual level to the material behavior of wood and its re-usability. It is a digital approach to a new timber-only joint typology, which investigates contemporary forms of cooperative multi-robotic processes and human–robot collaboration. The cooperation of two robotic arms allows a timber slat to be positioned in space by one arm while the other arm drills the holes for the dowels at a defined angle (Fig. 6c). These holes serve as a guide for easy manual insertion of the dowels and for an intuitive collaboration between students and robots. Taking advantage of the hygroscopic behavior of wood, the dowels are dried in an oven to reduce their diameter and thus further facilitate their insertion into the pre-drilled holes. Once moisture is then applied, their volumetric expansion maximizes the frictional force between dowels and slats, eliminating the need for any additional nails, glue or screws [25]. The different orientations of the connecting dowels mechanically lock the slats in their spatial position (Fig. 6a). Based on a simple geometric principle, this technique profits greatly from computational design and robotic fabrication to realize a variety of differentiated joint types.

Both Timber Dowel Assemblies projects involved working with external partners and clients, which introduces a real world context to the project and thus a real test for the application of advanced digital methods. Students have to consider a specific site and its constraints, a design brief, and institutional framework of the client, as well as the logistics involved in realizing the design and its segmentation, including transport, assembly, execution planning and delivery schedules. The design of the two large-scale projects presented here illustrates how the constructive system adapts to different contexts and constraints.

In the Gradual Assemblies project, as the curve radius of the design differs on the inner and outer side of the structure, an algorithmic branching logic within the computational design model allows to geometrically remove or add elements at strategic moments and to optimise the distribution of all timber slats (Fig. 4b). While the structure is robotically prefabricated in 22 larger segments, pre-drilled holes at the seams allow for a unique connection detail on-site: After the segments are placed in their respective position, they are connected by manually inserting dowels, which means the on-site connection detail is identical to the robotically fabricated one, leading to a monolithic and seamless character of the final structure that underlines the permanent character of the design (Fig. 6b).

In the Up Sticks installation, the design is based on a continuous surface built up by segments that twist, align and connect to form tubular bridging structures and spatial enclosures (Fig. 5b), so the computational design logic aims to maximize the possible curvatures of the system through the controlled spacing of the dowels. Additional dowels are generated to increase the structural capacity when the distances between individual slats become too wide or stresses within a joint become too great. The context of an exhibition also demands a design-for-disassembly approach, which is directly addressed by creating seams that can be geometrically adjusted, allowing for a flat contact face between the large segments that is tightened through screws that can easily be released.

#### Teaching strategy and learning experience: development of a performative joint typology

The performance of the joint and its geometric limitations play a central role in the constructive system of Timber Dowel Assemblies. As such, the type of joint becomes an important element, defining the design space by introducing structural as well as fabrication constraints. Therefore, the development of a performative joint typology becomes an important teaching goal that was addressed and iterated in each of the different teaching projects. The optimisation of the joint is a complex problem, as there are infinite possible combinations of dowel-slat connections, and every combination brings its own constraints. For each project, students thus have had the opportunity to weigh multiple objectives and to develop the joint typology in parallel with the design idea, the implementation of the fabrication process, and the evaluation of its structural performance (Fig. 6).

As such, a first joint type is explored in Gradual Assemblies, where each individual timber slat is directly connected to its neighbors by two oriented dowels. This facilitates the fabrication process as it reduces the number of dowels needed as well as their average length, allowing for precise in-place drilling and easy manual insertion of the dowels. Building upon this, another promising joint type is developed through an MAS Master Thesis. Here, the dowels extend to always connect three consecutive timber slats instead of only two, thus ensuring a better distribution of forces within the dowels and a stronger joint. This joint type is chosen for its structural performance, which is implemented and further developed in Up Sticks. In that project, the refined joint detail is combined with the closed ring geometries of the design, thus greatly increasing the overall stiffness of the structure. However, the fact that now each dowel has to connect three timber slats instead of two poses some challenges for the fabrication process. Instead of drilling the holes in-place, now all timber slats are robotically pre-drilled before being placed, requiring an extra level of precision in the placement to secure the alignment of three oriented holes for the insertion of the connecting dowels.

#### Discussion

Timber Dowel Assemblies demonstrates the development of a digital approach to timber-only connections, from first ideation to a performative system that can be further extended. Parts of the findings on the use of dowels for timber-timber joints was later applied to a large-scale construction project of Gramazio Kohler Research. The process itself challenges the classic divide between digital and analog processes, machine and craftsmanship. Lastly, given the limited resources on our planet, this project illustrates how contemporary knowledge in computation and digital fabrication technology can be combined with traditional knowledge about construction methods in order to create innovations in the construction sector.

### Rapid Clay Formations: UIA 2020 pavilion and Clay Rotunda

Rapid Clay Formations is a radical demonstration of how new construction methods based on natural material systems and robotic fabrication processes are developed from scratch through multiple teaching formats, gradually scaled and refined, and ultimately lead to the erection of a permanent building structure. Rapid Clay Formations explores a novel fabrication process for malleable materials, where soft clay cylinders are robotically picked, precisely positioned and pressed against each other to form a soft-bond through cohesion, plastic deformation and interlocking.

Compared to previous Integrated Design and Robotic Fabrication Projects, this project presents two new challenges: First, using a non-homogeneous material system such as a clay-based mix in combination with digitally controlled design and fabrication, this project requires that students address both complex material behavior and the need for geometric precision. Here, it becomes even more essential to combine empiric testing and prototyping with the development of predictive design tools, introducing the need for an adaptive design and fabrication workflow up to and during the fabrication phase. Second, shifting from a pre-fabrication and assembly to an on-site semi-mobile fabrication method changes the robotic control requirements as well as the logistics for planning and executing the construction. In addition to posing these two concrete challenges, the project addresses current global challenges for which the use of alternative construction materials could promote a sustainable and reusable building practice.

**Fig. 7:**
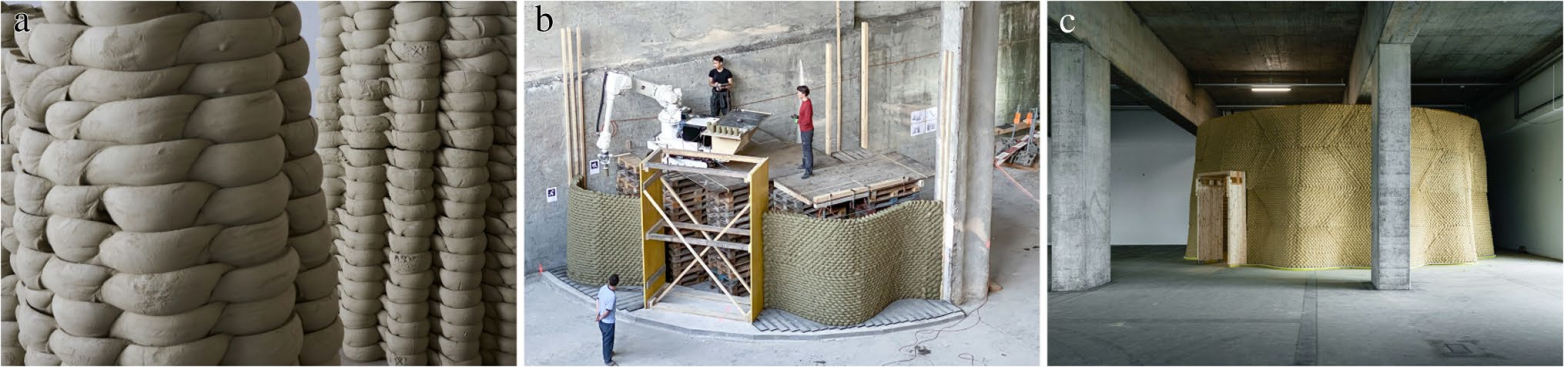
Rapid Clay Formations: Design development **a**) full-scale prototype for the UIA Pavilion **b**) mock-up of the Clay Rotunda, **c**) final Clay Rotunda

The concept of this constructive system originated with the Remote Material Deposition installation [5]. In that project, Gramazio Kohler Research investigated the idea of robotically positioning material in space from a distance, thereby creating differentiated architectural aggregations that are a direct expression of a dynamic and adaptive fabrication process. The concept was further developed within the MAS through multiple small-scale design and building exercises and a series of MAS Thesis projects. One new research idea that emerged from these early inquiries is the concept of Impact Printing, a new additive manufacturing method that aggregates malleable discrete elements (or soft particles) by a robotic shooting process [8, 22]. Continuing this concept, more recent projects developed within the MAS programme shifted the focus to the study of robotic pressing processes for malleable materials, where the precise control of forces and positions applied to the material allows the students to design and build highly differentiated structures [7, 9]. The project we discuss here is the first architectural scale implementation of the fabrication process for an Integrated Design and Robotic Fabrication Project, the UIA 2020 Pavilion (Fig. 7a) [12]. The Pavilion was intended to be built for the World Congress of Architects in Rio de Janeiro but could not be realised due to the Covid-19 outbreak, so instead the process and application were further developed for a large-scale mock-up (Fig. 7b) [13] and a permanent structure in Switzerland, the Clay Rotunda (Fig. 7c) [14].

#### Material system, fabrication process, and design

**Fig. 8:**
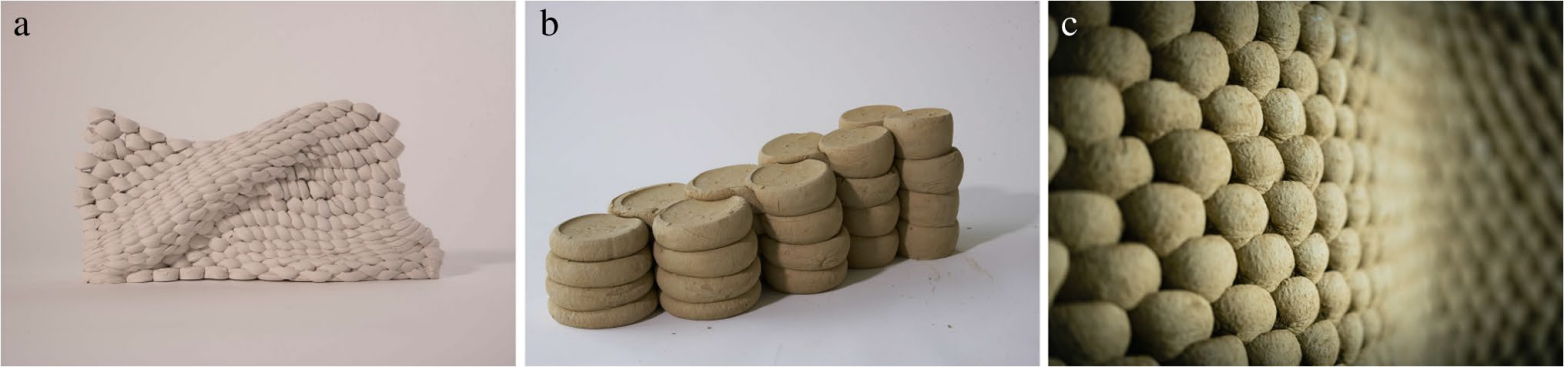
Rapid Clay Formations: Developing the Tectonics of a Soft-Bond **a**) small-scale prototype with differentiation of force applied **b**) full-scale prototype for the UIA Pavilion **c**) running soft-bond for the Clay Rotunda

The constructive system can be described as a contemporary reinterpretation of earthen construction, where unfired cylinders of clay, which we call soft-bricks, are robotically aggregated to form what we describe as a soft-bond (Fig. 8). Each individual soft-brick is robotically picked by a custom gripper, precisely oriented and positioned, released and then pressed against one the others in a quasi-layered building process. Taking full advantage of the malleability of the material in its wet state, the compressed cylinders form a strong connection through surface cohesion and geometric interlocking. The unique material expression is created by the plastic deformation of the soft-bricks and defines a completely novel aesthetic, at the interplay of a digital control and the complexity of a non-homogeneous material behavior. In contrast to dealing with discrete solid elements such as fired bricks, working with a malleable material system means that both the segmentation and sequencing influences the geometric outcome of the process.

The initial design for the UIA 2020 Pavilion resulted in the proposal for an undulating clay wall with a length of approximately 21 m and up to two meters high. Findings on the material mix and processing, the soft-bond typology and its segmentation, as well as the control of the robotic fabrication process formed the base for the design development of the Clay Rotunda, a freestanding, single layer earth-based cylindrical structure constituting the outer, soundproof shell of a high-fidelity music auditorium built inside a former Brewery in Bern (Fig. 7c).

Following an open design brief, the original proposal for the UIA pavilion envisioned a curvilinear wall that creates specific spatial conditions in relation to its site and context. For this purpose, students developed the design synchronously with the material and structural system through explorations at different scales. They developed a differentiated soft-bond, optimising the structural behaviour of the system so as to be able to build up to two meters in height. As the performance of the process is highly dependent on the material behavior, the design and robotic process are informed by the continuous refinement of a suitable material mix, which was optimized with the main goal of reducing the shrinkage and chemical cracks while still retaining enough plasticity to ensure proper bonding between the elements and reach considerable heights. The findings from the pavilion project were then integrated into the design development for the Clay Rotunda. The clay mix was further optimized to increase the structural capacity and bonding behaviour of the soft-bricks, which made possible the impressive scale of the structure. It features a diameter of almost 11 m and is 35 m in length, and it reaches a height of 5 m with a width of just 15 cm in unreinforced clay, with 30′000 clay cylinders in total.

#### Teaching strategy and learning experience: developing the tectonics of a soft-bond

The development of a suitable soft-bond typology is key for the design explorations with Rapid Clay Formations. The design process has to pull together different aspects of the constructive system, including the material behaviour and its expressive aesthetic, the robotic fabrication process, and the structural capacity. Physical testing at different scales is necessary to understand the complex behaviour of the malleable material system in relation to the precise control by computational design and digital fabrication.

The goal of a first short-term design research and fabrication exercise as part of the first trimester Digital Foundations curriculum was to explore the typological and structural potential of the soft-bond at small-scale. Topics included varying force applied to the soft-bricks to create different bonding behavior and thickness of the structure, varying soft-brick sizes (diameter and height) and their respective interlocking behavior, discretizing surfaces into single and multilayer high-resolution aggregations, and exploring the possibility of different soft-brick shapes (Fig. 8a) [9].

For the first application at full architectural scale for the design proposal of the UIA Pavilion, the development of the soft-bond aimed to allow for differentiated expression and high structural performance while minimizing the material used. At the bottom part of the wall, the cylinders are assembled in a stacked-bond that doubles-up to provide the necessary structural width, while at the top section of the wall the topology transitions to a running-bond, creating a thin and elegant expression (Fig. 8b). Maintaining structural integrity was one of the main challenges; while earthen structures usually depend on large cross-sections and heavy material masses, we aimed to minimize the amount of material to reduce the construction time and ensure rapid drying, as well as to improve material ecology [12].

**Fig. 9:**
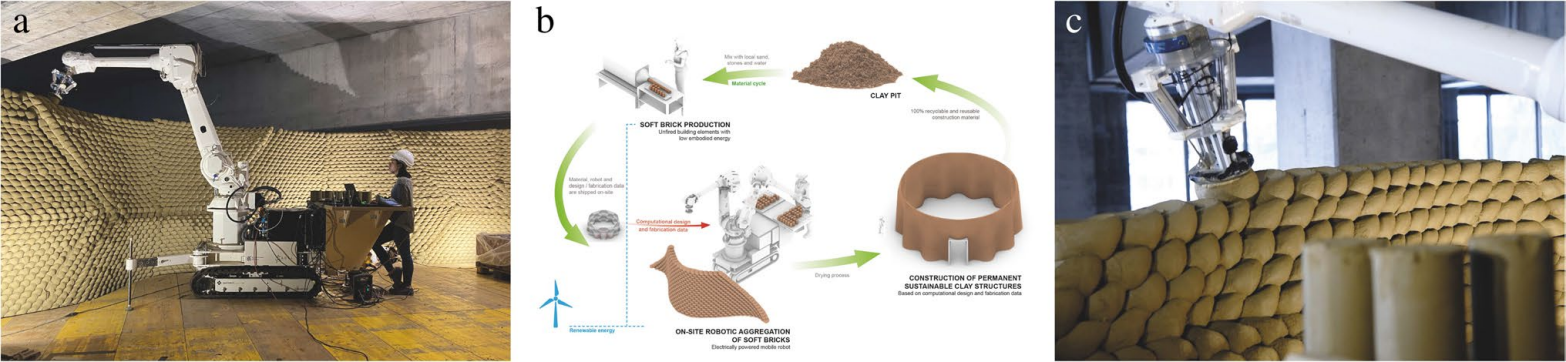
Rapid Clay Formations as a sustainable construction process **a**) on-site robotic fabrication with a malleable material **b**) circular use of material **c**) close-up of the robotic pressing process

When the project was stopped due to the Covid-19 outbreak, the soft-bond concept was further developed for the subsequent realization of the Clay Rotunda, realised using the mobile robotic platform In-situ Fabricator (Fig. 9a) [6, 15]. The extreme slenderness of the Clay Rotunda is made possible by its undulated design, which increases the footprint and stabilizes the structure in order to prevent buckling effects (Fig. 8c). The limited reach of the robotic arm and the shrinking of the material while drying demanded complex strategies to decide how to segment the structure horizontally and vertically into matching trapezoids. The specific orientation in which the robotic arm pressed onto to the seams in between these trapezoids was particularly important to ensure proper bonding of the different segments (Fig. 9c) [14].

#### Discussion

The work on this project contributed to a successful application for a large research grant to continue our investigations beyond the teaching format and to the first large-scale implementation of the soft-bond in practice. Thus, the project overall has acted as a catalyst for knowledge transfer between research and industry. During these explorations, a smaller scale robotic setup allowed for quick prototyping at model scale to explore the typological and topological variations of soft-bonds and their design potential. At the same time, the large mobile robotic platform allowed us to build full-scale prototypes to ensure the scalability of the process and to investigate the material and structural performance of the system over time. Combining the traditional knowledge of clay constructions with contemporary digital design and fabrication processes, the robotic clay aggregation process increases the degree of control of both material and process, allowing complex structures to be built that go beyond what has traditionally been possible. The process pushes the usage of locally sourced clay, suggesting a meaningful and sustainable approach to robotic construction of earthen structures. Finally, as minimizing energy consumption and emissions becomes more important in the context of digital fabrication, Rapid Clay Formations proposes an entirely waste-free, fully reversible and recyclable constructive system, promoting the circular use of material from “pit-to-pit” (Fig. 9b).

## Conclusions and outlook

### Conclusions

The Integrated Design and Robotic Fabrication Project is highly valuable not only as a teaching format but also as a catalyst to explore new research topics. While it may seem ambitious for the projects to design, develop, and deliver large-scale structures in an experimental setup in only 10 weeks, this short duration encourages a culture of intense collaboration and interdisciplinarity throughout the whole process. This fosters skills that cannot be taught ex-cathedra or in separate manner, but which students, tutors and project partners alike can acquire through this all-encompassing experience. We argue that understanding the building format as a laboratory for experimentation allows us to question and rethink conventional design approaches to material and constructive systems while at the same time critically assessing computational design and digital fabrication strategies — strategies that are often celebrated as innovative without further scrutiny. Although all case studies discussed relate to and build upon prior research investigations, they all take a radical stance towards topics such as alternative material systems, sustainability, reusability, detailing and construction planning. As such, many of the ideas tested within the MAS Integrated Design and Robotic Fabrication Project have opened up new research streams that have been continued in different formats. This close relationship of research and teaching topics is rooted in the understanding of the Integrated Design and Robotic Fabrication Project as a form of collaborative design research. From a pedagogical perspective, with the MAS being a post-graduate programme in the rather emerging field of digital fabrication, this integration of research within teaching allows students to develop as independent young professionals.

In summary, the projects cannot be characterized as pure research demonstrators, large-scale prototypes or “student pavilions”. Instead, they reflect that we are addressing a larger ambition, namely the development of a new practice of Architecture. Shifting from the Albertian paradigm of the designer author as an idealist-intellectual, disconnected from the actual task of building [3], we emphasize a methodology which addresses the complexities and challenges of today’s design and building processes, from digital information and physical material to the construction and life-cycle of a building. By exploring technology in the context of an actual building task, computational design and digital fabrication skills are developed and tested on meaningful questions such as the need for more sustainable material processes, the concepts of reversibility, recycling and reuse. Acknowledging the architect’s need to comprehend both the big picture and the specialist perspective, this endeavor suggests a new idea of authorship in the form of an interdisciplinary and collaborative entity. In times where our global challenges ask for a re-evaluation of our building practice, the approach presented here offers a valuable contribution to the future of our discipline.

### Outlook

During the past four years, the Integrated Design and Robotic Fabrication Project has evolved through the development of novel computational design and robotic fabrication techniques. The evolution went from the assembly of traditional construction materials following a conventional structural system (The Brick Labyrinth) to traditional materials fabricated into novel spatial construction systems (Timber Dowel Assemblies) and ventured most recently into the spatial aggregation of malleable materials with complex behaviour (Rapid Clay Formations). This rapid growth in complexity enabled the development of novel computational design methods, material simulation and adaptive robotic fabrication methods, but increased the necessary time for preparation. Here, we push for a conceptual change, where instead of developing novel processes, research topics which have reached a certain level of maturity are integrated as the base for the teaching agenda. The approach allows researchers, tutors and students to experience research as a form of architectural practice. Our project is based on the idea that students can be part of investigating how digital technologies will reform the way we design and build, and this participation forms the foundation of our pedagogy for training future architects.

## Footnotes

1 With the hygroscopic behaviour of wood, dowels shrink and swell according to their moisture content. Here, this volumetric expansion maximizes the frictional force of the dowel connection.
